# *BAP1* Malignant Pleural Mesothelioma Mutations in *Caenorhabditis elegans* Reveal Synthetic Lethality between *ubh-4*/*BAP1* and the Proteasome Subunit *rpn-9*/*PSMD13*

**DOI:** 10.3390/cells12060929

**Published:** 2023-03-18

**Authors:** Carmen Martínez-Fernández, Sweta Jha, Elisabet Aliagas, Carina I. Holmberg, Ernest Nadal, Julián Cerón

**Affiliations:** 1 Modeling Human Diseases in *C. elegans* Group, Genes, Diseases, and Therapies Program, Institut d’Investigació Biomèdica de Bellvitge (IDIBELL), L’Hospitalet de Llobregat, 08908 Barcelona, Spain; 2 Medicum, Department of Biochemistry and Developmental Biology, Faculty of Medicine, University of Helsinki, Haartmaninkatu 8, 00290 Helsinki, Finland; 3 Department of Medical Oncology, Institut Català d’Oncologia (ICO), L’Hospitalet de Llobregat, 08908 Barcelona, Spain; 4 Preclinical and Experimental Research in Thoracic Tumors (PReTT), Institut d’Investigació Biomèdica de Bellvitge (IDIBELL), L’Hospitalet de Llobregat, 08908 Barcelona, Spain

**Keywords:** *C. elegans*, *ubh-4*, BAP1, *rpn-9*, PSMD13, Malignant Pleural Mesothelioma, Bortezomib, CRISPR-Cas, proteasome

## Abstract

The deubiquitinase BAP1 (BRCA1-associated protein 1) is associated with *BAP1* tumor predisposition syndrome (TPDS). *BAP1* is a tumor suppressor gene whose alterations in cancer are commonly caused by gene mutations leading to protein loss of function. By CRISPR-Cas, we have generated mutations in *ubh-4*, the *BAP1* ortholog in *Caenorhabditis elegans*, to model the functional impact of *BAP1* mutations. We have found that a mimicked *BAP1* cancer missense mutation (UBH-4 A87D; BAP1 A95D) resembles the phenotypes of *ubh-4* deletion mutants. Despite *ubh-4* being ubiquitously expressed, the gene is not essential for viability and its deletion causes only mild phenotypes without affecting 20S proteasome levels. Such viability facilitated an RNAi screen for *ubh-4* genetic interactors that identified *rpn-9*, the ortholog of human PSMD13, a gene encoding subunit of the regulatory particle of the 26S proteasome. *ubh-4*[A87D], similarly to *ubh-4* deletion, cause a synthetic genetic interaction with *rpn-9* inactivation affecting body size, lifespan, and the development of germ cells. Finally, we show how *ubh-4* inactivation sensitizes animals to the chemotherapeutic agent Bortezomib, which is a proteasome inhibitor. Thus, we have established a model to study *BAP1* cancer-related mutations in *C. elegans*, and our data points toward vulnerabilities that should be studied to explore therapeutic opportunities within the complexity of *BAP1* tumors.

## 1. Introduction

*BRCA1-associated protein-1* (*BAP1*) was identified as a tumor suppressor gene in 2008 [1]. Germline pathogenic variants in *BAP1* are associated with a variety of tumors in the context of the *BAP1*-tumor predisposition syndrome (TPDS) characterized by familial occurrence of cutaneous melanocytic tumors, uveal melanoma, malignant mesothelioma of the pleura and the peritoneum, renal cell carcinoma and specific non-malignant neoplasms of the skin [2]. In a study of 350 patients affected by BAP1-TPDS, 84.3% inherited a *BAP1* mutant allele and developed one or more malignancies during their lifetime [3]. Thus, although most *BAP1* mutations are germline mutations, somatic mutations also occur. A genomic study identified somatic inactivating *BAP1* mutations in 23% of the 53 MPM tumor samples analyzed [4]. In our study, we have mimicked two of the somatic mutations cited in that article (BAP1 F81V and A95D). Since cancer alterations in *BAP1* are typically caused by genetic mutations leading to protein loss of function [5,6], we have also generated deletion alleles of its *ubh-4 C. elegans* ortholog.

Malignant Pleural Mesothelioma (MPM), the most common type of malignant mesothelioma, is particularly aggressive, affecting about one and four individuals in 100,000 people in the US and UK, respectively [7]. The only FDA-approved first-line treatment regimen for patients with advanced, unresectable MPM combines cisplatin with either pemetrexed or raltitrexed [7,8]. Cisplatin plus pemetrexed has been the standard of care treatment for several decades and the addition of bevacizumab, an antibody for a vascular endothelial growth factor (VEGF), modestly improved the overall survival [9]. More recently in the CheckMate-743 trial, frontline dual immune checkpoint blockade with nivolumab plus ipilimumab improved the overall survival compared with platinum-based chemotherapy [10]. This immune checkpoint combination became the new standard of care in many countries. However, only a minority of patients with MPM are long-term survivors and novel therapeutic approaches are needed.

BAP1 is part of the ubiquitin proteasome system and encodes a deubiquitinase (DUB) that has a Ubiquitin Carboxy-terminal Hydrolase (UCH) domain and nuclear localization signals (NLS) that are essential for its nuclear function [11]. BAP1 received its name because it is a BRCA1-associated protein. Although several functional interactions have been described for these two proteins [12,13], it is still controversial whether BAP1 and BRCA1 (the latter being a tumor suppressor gene with a ubiquitin E3 ligase activity) physically interact with each other. BAP1 has been associated with diverse nuclear and cytoplasmic functions including cell death regulation, cell pluripotency maintenance, DNA damage response and replication, cell cycle progression, histone modification, and modulation of metabolism [14,15,16,17,18,19].

Given the conservation of this protein in *C. elegans* ortholog *ubh-4*, we built an in vivo model to study *BAP1* inactivating mutations and to gain insight into BAP1 potential interaction with other factors. The efficiency and speed of CRISPR-Cas in *C. elegans* can help to mimic human *BAP1* mutations in nematodes to better understand BAP1-TPDS and its complex genetic landscape, and ultimately to unveil drug targets for the development of new treatments for MPM patients with *BAP1* mutations.

## 2. Materials and Methods

### 2.1. Caenorhabditis elegans Strains

*C. elegans* strains were maintained using standard procedures [20]. Before conducting the experiments, strains were grown for at least two generations at the experimental temperature. Worms were synchronized using sodium hypochlorite [21]. N2 was used as a wild-type strain. Used strains for this study were generated by CRISPR-Cas or provided by the Caenorhabditis Genetics Center (CGC) and genotyped by PCR (Supplementary File S2).

### 2.2. CRISPR Generation of Strains

Guide RNAs were designed using both Benchling and CCTop online tools. CRISPR-Cas9 mutants and reporter strains were obtained following a co-CRISPR strategy [22] using *dpy10* as a marker to enrich for genome-editing events [23]. RNP complexes containing crRNA, tracrRNA, and commercial Cas9 (IDT) were annealed at 37 °C for 10 min and later, the remaining reagents were added. CRISPR mixes were centrifuged for 5 min at 13,000 RCF and injected into the gonads of young adult hermaphrodites using the XenoWorks Microinjection System. F_1_ progeny was screened by PCR using specific primers and F_2_ homozygotes were confirmed by Sanger sequencing. crRNAs, repair templates and the composition of the injection mixes are specified in a supplementary excel file (Supplementary File S3).

### 2.3. Body Length

A synchronized population of L1-arrested larvae was cultured on NGM plates containing fresh OP50 and 60 µg/mL of cisplatin. The body length of ≥50 worms for each condition was measured at 72 h or 96 h at 20 °C on the stereomicroscope using the NIS-Elements 3.2 imaging system. Experiments were conducted at 1 °C, the body length was measured after 5 days of incubation. Each assay was done in duplicate, and at least two biological replicates were performed.

### 2.4. Brood Size

A synchronized population of L1-stage worms was grown into NGM plates, with fresh OP50 until L4 stage at 20 °C. An average of 12 animals from each genotype were seeded individually into separated plates. Animals were passed to a new plate after few days until total progeny were laid; progeny was counted after each pass.

### 2.5. RNA Interference

For the RNAi screen, *ubh-4* knockout (*cer27*) was used and N2 as the wild-type strain. A 150-gene sublibrary was generated to perform the screen. RNAi clones were obtained from the ORFeome library [24] or the Ahringer library [25] and insert size was validated by PCR. The classification and description of these genes are collected in Supplementary File S1. Bacterial cultures of different RNAi clones were grown overnight at 37 °C in LB with 50 µg/mL ampicillin and 12.5 µg/mL tetracycline. RNAi 24 multiwell plates (NGM media was supplemented with 3 mM IPTG, 50 µg/mL ampicillin, and 12.5 µg/mL tetracycline) were seeded with 30 µL of bacterial cultures of different RNAi clones. dsRNA synthesis was induced overnight at room temperature. Synchronized L1-arrested worms were placed onto RNAi plates and different phenotypes were scored at every day at 20 °C for 168 h. The screen was conducted in triplicate for each condition and was performed two different times, using fresh batches of RNAi plates and bacterial cultures. In all the cases, a *gfp(RNAi)* clone was used as a negative control. Hits were further validated in 55 mm RNAi plates seeded with 300 µL the corresponding RNAi clone. For *rpn-9* deletion mutants, individual animals were plated separately when they reached L4 stage and were passed into a new RNAi plate every two days until they stopped laying eggs. Hatched larvae, dead embryos and animals presenting cuticle abnormalities were scored.

### 2.6. Bortezomib Treatment and Survival Assay

Animals at the L4 stage were picked to NGM seeded plates containing Bortezomib (Merck KGaA, Darmstadt (Germany), Cat# 5043140001) at concentrations of 5 µM, 10 µM, 15 µM, 20 µM or 30 µM, or DMSO in corresponding volumes. Animals were closely monitored after 24 h for three days and scored at 1-day, 2-day, and 3-day intervals of adulthood as normal, sick (defective movement and appearance), very sick (highly defective movement and appearance) and dead.

### 2.7. Immunofluorescence with Dissected Animals

Age-synchronized 1-day adult animals were harvested in an M9 buffer. Animals were transferred onto a glass dish and immobilized prior to dissection using 1 mM levamisole hydrocloride (Merck KGaA, Darmstadt, Germany, Cat# T7660). Incisions with a 27-gauge syringe needles were made close to the pharynx forcing the intestine and gonad to extrude. Dissected animals were fixed with 2×RFB (160 mM KCL, 40 mM NaCl, 20 mM EGTA, 10 mM spermidine, 30 mM PIPES pH 7.4, 50% methanol, and 1% formaldehyde), followed by a short 100% methanol fixation. The fixed dissected animals were permeabilized with 0.5 or 1% Triton X-100 in PB before mounting with SlowFade Diamond Antifade Mountant (Thermo Fisher Scientific, Waltham, MA, USA, Cat# S36967). The antibodies against the proteasome 20S alpha subunits (Enzo Life Sciences, New York, NY, USA, Cat# BML-PW8195,) were used in 1:200 dilution. Alexa fluor 594 conjugated anti-mouse IgM (Thermo Fisher Scientific, Cat# A-21044) and IgG (Thermo Fisher Scientific, Cat# A-11005, or Cat# R37121) secondary antibodies in 1:100 dilution were used for visualization. DNA was stained with 4 ug/mL Hoechst 33342 (Merck KGaA, Darmstadt, Germany, Cat# B2261).

### 2.8. Immunohistochemical Analysis

Age-synchronized 1-day adult animals were collected in M9 and fixed with 10% (*v*/*v*) phosphate-buffered formalin. Formalin-fixed animals were embedded in 2% agar, and paraffin-embedded agar blocks were cut into 4 µm sections. Immunohistochemical staining was performed using Dako REAL^TM^ EnVision^TM^ Detection System, Peroxidase/DAB+, Rabbit/mouse kit (Agilent Dako, Santa Clara, CA, Cat# K500711-2) and the slides were immunostained using the anti-20S alpha antibody (Enzo Life Sciences, BML-PW8195) in 1:1000 dilution following the protocol previously reported [26].

### 2.9. Graph Plotting and Statistical Analysis

Data representation and statistical analysis were performed using GraphPad Prism 8.

## 3. Results

### 3.1. ubh-4 Is the C. elegans Ortholog of Human Proteins UCHL5 and BAP1

**Figure 1:**
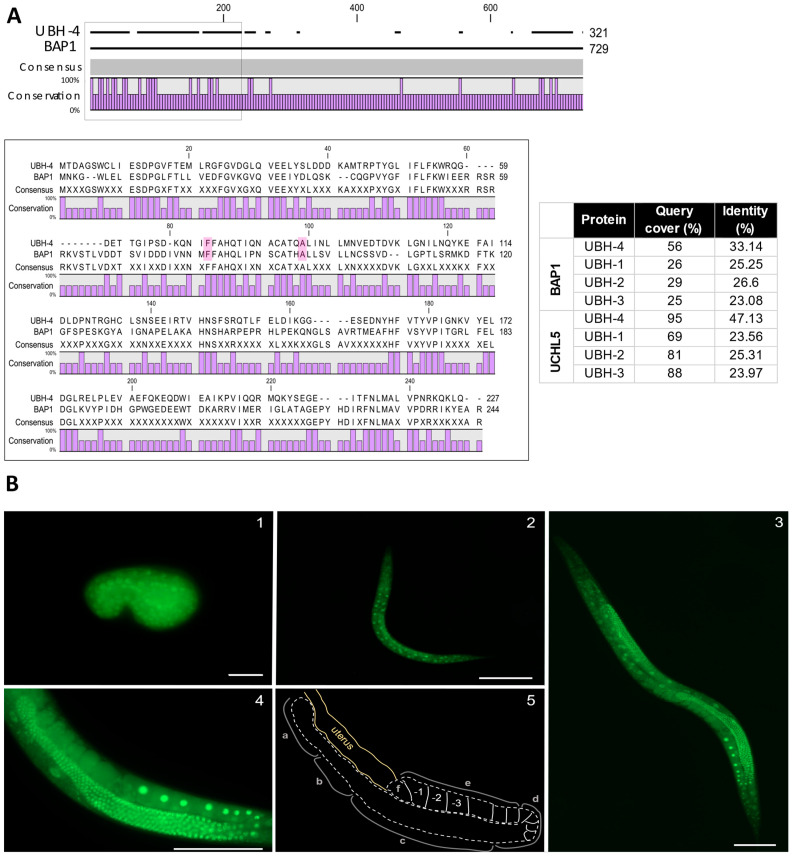
*C. elegans* BAP1 ortholog UBH-4 homology and expression pattern. (**A**) UBH-4 is the ortholog of human BAP1. Scheme of UBH-4, UCHL5 and BAP1 protein sequences (black lines) and conserved residues (purple bars), predominantly at the N-terminal and C-terminal domains. In the black square are the catalytic domains of these proteins. The bottom scheme shows with more detail conserved residues between UBH-4 and BAP1. Pink shadows indicate MPM-related residues modelled in this study. Table shows BLASTP analysis of *C. elegans* UCHs proteins, UCHL5 and BAP1. Alignments were illustrated by CLC Sequence Viewer 8.0. (**B**) UBH-4 is ubiquitously expressed during *C. elegans* development. Representative images of UBH-4::EGFP signal in developing embryo (1), L3 (2), and adult stages (3). Scale bars represent 10, 100, and 100 µm, respectively. (4) Posterior arm of the adult gonad at higher magnification (scale bar represents 100 µm) and its diagram. (5) (2) pointing out distal mitotic (a), transition (b), and meiotic zones (pachytene (c), diplotene (d) and diakinesis (e)), and spermatheca (f).

Sequence similarity findings indicate that UBH-4 is the ortholog of human UCHL5 and BAP1, sharing 47.13% and 33.14% identity with these two proteins, respectively (Figure 1A) [27]. The two proteins belong to the Ubiquitin C-terminal Hydrolase (UCH) family, which is part of the Ubiquitin Cysteine Peptidase C12 superfamily. UCHL5 and BAP1 contain an N-terminal catalytic domain with cysteine peptidase activity, which is conserved in UBH-4 (Figure 1A). The C-terminal region, including the C-terminal Binding Domain (CTD) and Nuclear Location Signals (NLSs), also present a high level of conservation among the three proteins. The C-terminal end of BAP1 and UCHL5 is involved in interactions with other proteins and regulatory functions [28]. Thus, given the identity at the amino acids level, it is expected that UBH-4 performs the ancestral functional roles of UCHL5 and BAP1, pointing *C. elegans* as a suitable model for studying the effect of mutations in *BAP1* orthologues.

### 3.2. ubh-4 Is Ubiquitously Expressed in All Developmental Stages

First, to investigate the cellular and subcellular distribution of UBH-4, we generated an endogenous fluorescent reporter for *ubh-4* by Nested CRISPR [29]. We observed ubiquitous UBH-4 expression at physiological levels in all developmental stages (Figure 1B). In the hermaphrodite *C. elegans* germline, the proliferative region (mitotic zone) is at the distal part, followed by a meiotic region with cells at different meiotic stages until they form oocytes, which are self-fertilized after crossing the spermatheca. Interestingly, UBH-4::EGFP in the adult germline displays a more intense fluorescent signal at the meiotic region, suggesting a relevant role for UBH-4 in meiotic progression (Figure 1B).

### 3.3. Mimicking Human BAP1 Mutations in C. elegans ubh-4

**Figure 2:**
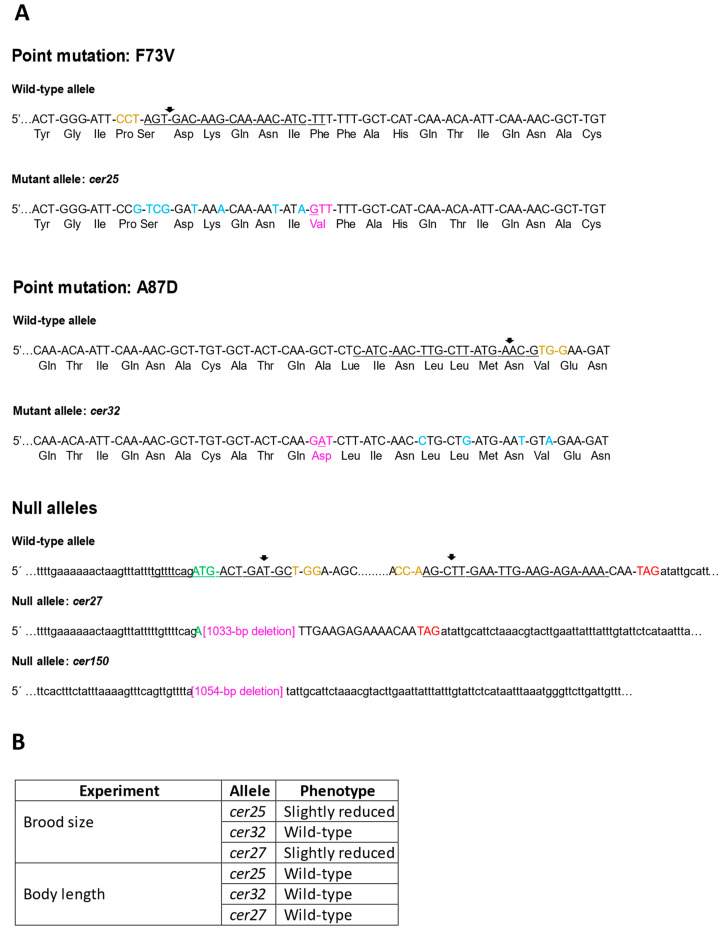
CRISPR-Cas generation and characterization of UBH-4 mutant alleles. (**A**) Molecular designs to generate the *ubh-4* cancer-related alleles *cer25* and *cer32* by CRISPR-Cas9. Sense strands are represented. PAM sequences are shown in yellow; crRNA sequences are underlined and Cas9 cut sites are indicated by black arrows; silent mutations are labelled in light blue; mutated codons are shown in pink; nucleotide changes (T > G (*cer25*) and C > A (*cer32*)) are underlined. Aminoacidic sequences are represented below nucleotide sequences, highlighting the mutated residues in pink. (**B**) Phenotypes observed for indicated alleles after brood size, body length and proteasome amount and activity characterization.

Thanks to the high level of conservation between BAP1 and UBH-4 at the N-terminal, we mimicked *BAP1*-like cancer-related missense mutations in *C. elegans* to explore their functional impact on this pluricellular organism. We created mutations affecting two residues close to C91, a residue functionally relevant at the active site [1], which are also conserved in *C. elegans* UBH-4 (Figure 1A), and correspond to a functionally relevant residue for BAP1 activity [2,3]. These *BAP1* cancer-related missense mutations were p.F81V and p.A95D, which correspond to *C. elegans* p.F73V and p.A87D, respectively. The CRISPR-Cas genome editing in *C. elegans* (Figure 2A) resulted in animals with genotypes *ubh-4*(*cer25*[F73V]) and *ubh-4*(*cer32*[A87D]) that do not display any overt phenotype.

Then, since other *BAP1* somatic mutations include frame-shift mutations that may result in the inactivation of BAP1 functions [4], we investigated the potential consequences of UBH-4 loss-of-function by producing null alleles. The *ubh-4* gene produces two transcripts, C08B11.7.1 and C08B11.7.2, both with a 966 nucleotides coding sequence differing only at their 3′ UTR. We generated null alleles for *ubh-4* by non-specific NHEJ (non-homologous end joining) repair of a CRISPR-Cas9-induced DSB (allele *cer27*), and by HDR (homology-directed repair) using a repair template (allele *cer150*), resulting in the depletion of 1033 (*cer27*) and 1054 (*cer150*) base pairs, respectively (Figure 2A). As a result of this editing, both alleles lost their start codons and did not produce any transcripts (confirmed by RT-PCR). Therefore, these two *ubh-4* deletion alleles were considered null or without function. Surprisingly, despite *ubh-4* being expressed ubiquitously and throughout the *C. elegans* lifecycle, no overt phenotypes were observed, just a slight reduction of the progeny in animals harboring the deletion allele (*cer27*) and the missense mutation F73V(*cer25*) (Figure 2B). Accordingly, in the absence of penetrant phenotypes, we observed that mutations in *ubh-4* do not alter the 20S proteasome tissue levels (Figure S1), despite encoding a proteasome-associated DUB [30].

### 3.4. RNAi-Based Screen Reveals rpn-9 as Genetic Interactor of ubh-4

Since *ubh-4* null mutants were viable and did not display overt phenotypes, we wondered whether the lack of *ubh-4* function would influence the activity of other genes or genetic pathways. Thus, we performed an RNAi screen in *ubh-4(cer27)* mutants to identify synthetic genetic interactions with *ubh-4*. Using the two existing *C. elegans* RNAi libraries as sources of clones [24], we selected 150 RNAi clones whose identities were verified by PCR (by checking the size of the insert in each clone) (Supplementary Table S1). This RNAi sublibrary included clones that inactivate proteasome subunits, proteasome-related enzymes, orthologs of known and putative BAP1 interactors, genes frequently mutated in cancer, and malignant BAP1-TPDS-related genes [31,32,33,34]. We also added potential UBH-4-protein interactors according to information in the STRING 11.0 database [35].

**Figure 3:**
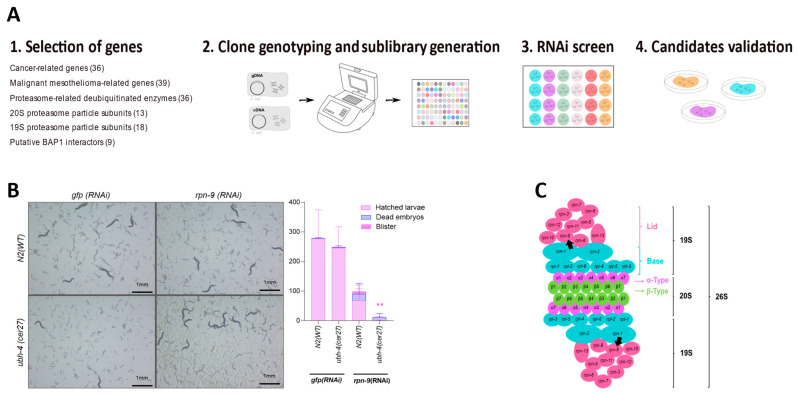
RNAi for synthetic lethal interactions with *ubh-4* mutants. (**A**) RNAi screen workflow is illustrated. After candidate genes are selected (1), clones from RNAi libraries were picked aleatorily and genotyped to generate a 150-gene RNAi sublibrary for screening (2). For the RNAi screen, 150-RNAi cultures were IPTG-induced in RNAi 24-well plates. Synchronized L1 animals were seeded and checked every day for 120 h at 20 °C, to score both wild-type and *cer27* animals (3). Finally, candidate genes were validated in larger (55 mm-diameter) RNAi plates (4). (**B**) Representative images of reduced brood size in *ubh-4(cer27)* background after 120 h *rpn-9(RNAi)* treatment (20 °C). The chart quantifies hatched larvae, dead embryos, and blister animals under different RNAi conditions. Statistics were performed by one-way ANOVA (Kruskal–Wallis and Dunn’s tests). ** *p* < 0.01 comparing hatched larvae between wild-type and *cer27* under *rpn-9(RNAi)*. No significant differences were found between the other observed phenotypes. An average of 20 animals were scored. The experiment was performed once. (**C**) Schematic model of the *C. elegans* 26S proteasome. 26S complex is formed by two regulatory particles 19S and one core particle 20S [39]. Structure subunit forms are represented by colors and detailed subunit proteins by coding gene names. Rings conforming to the 20S core and 19S structure are represented linearly. Black arrows point out *rpn-9* subunits. Illustrations were drawn using Inkscape 1.2.2. (Retrieved from https://inkscape.org).

The 150-gene RNAi library was screened in 24-well plates at 20 °C. Clones producing synthetic interactions were further validated in larger RNAi plates (Figure 3A). As a result, we concluded that RNAi inactivation of *rpn-9*, a non-ATPase subunit of the 19S proteasome (a regulatory particle of the 26S proteasome) [36], caused a robust and significant reduction of the brood size in *ubh-4(cer27)* animals (Figure 3B). Therefore, *rpn-9* was identified as an *ubh-4* genetic interactor. Since PSMD13, the RPN-9 human ortholog, is required for proteasome-mediated protein degradation [37,38] (Figure 3C), the proteasome subunit RPN-9 and UBH-4 may cooperate to regulate the degradation of ubiquitinated proteins.

### 3.5. BAP1 Cancer-Related Mutation A87D Mimics ubh-4 Deletion Phenotypes in rpn-9(gk140) Background

**Figure 4:**
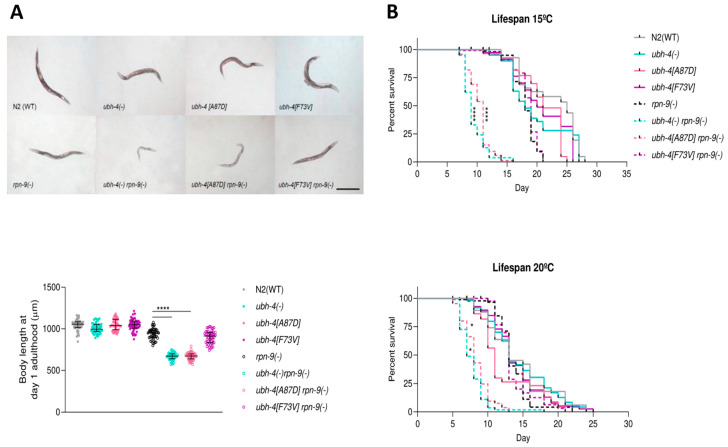
*ubh-4* deletion and [A87D] significantly reduces body length and survival in the *rpn-9* mutant background. (**A**) Representative images showing smaller body length in *ubh-4* and *rpn-9* single and double mutants (i, ii, iii, or use top and bottom rows). Pictures were taken at day 1 of adulthood growth at 1 °C. Scale bar represents 0.5 mm. Below, the chart quantifies the body length in wild-type (WT), single, and double mutants. Bars represent median and interquartile range, and dots the measured length of individual animals. The same experiment was performed three times, measuring an average of 100 animals per replicate. **** mean *p* < 0.0001. Statistics were analyzed by one-way ANOVA (Kruskal–Wallis and Dunn’s tests). (**B**) Survival curves of WT, *ubh-4*, and *rpn-9* simple and double mutants at 1 °C and 2 °C. Percent survival is indicated for each allele. An average of 80 animals were censored through two independent experiments for lifespan analysis at both temperatures. Statistical analysis by Log-rank (Mantel–Cox) test was performed to compare *rpn-9(gk401)* and double mutants. * and **** indicate *p* < 0.1 and *p* < 0.0001, respectively.

To validate the hit resulting from the RNAi screen, we studied the synthetic phenotype between *ubh-4* and *rpn-9* by using genetic mutants. Since *ubh-4* and *rpn-9* are located on chromosome II, we performed CRISPR-Cas9 gene editing in *rpn-9(gk401)* mutants to have both mutations in the same strain. *rpn-9(gk401)* is a deletion allele causing sterility in homozygosis, thus only heterozygous animals (balanced with the balancer chromosome mIn1 containing *myo-2::GFP* as a marker) were microinjected to generate double-mutant strains with the *ubh-4* deletion allele *(cer140)* and the *ubh-4* missense mutations *cer195*[*F73V*] and *cer198*[*A87D*]. We observed that although *rpn-9(gk401)* mutants present a reduced body length compared with wild-type animals, this *rpn-9* mutation produces even smaller animals in the *ubh-4(cer140)* and *ubh-4(cer198[A87D])* backgrounds (Figure 4A).

Since adult animals harboring the *rpn-9(gk401)* in combination with *ubh-4(cer140)* and *ubh-4(cer198[A87D])* seemed sicker, we monitored their lifespans at 15 °C and 20 °C. Interestingly, there was a dramatic decrease in these double-mutant strains compared to wild-type and the double-mutant *rpn-9(gk401)*; *ubh-4(cer195[F73V])* (Figure 4B). This decrease was more abrupt at 15 °C than 20 °C. These two described phenotypes suggest that the missense mutation *ubh-4(cer198[A87D])* is a loss-of-function mutation that phenocopies the *ubh-4* deletion allele.

### 3.6. UBH-4 and RPN-9 Present Ubiquitous and Overlapping Expression Patterns

**Figure 5:**
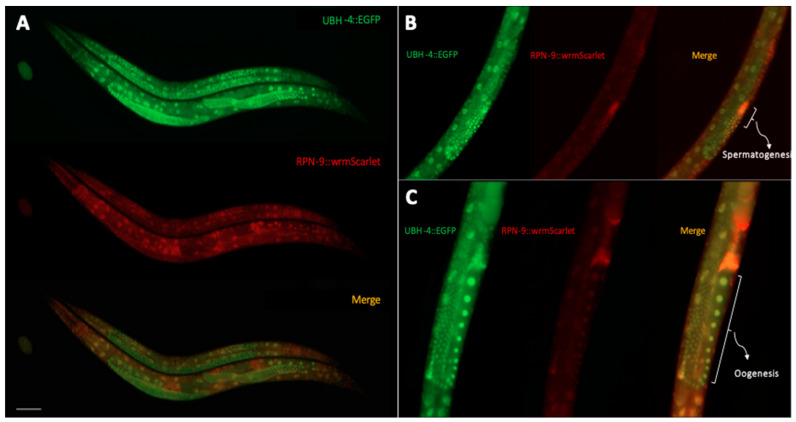
UBH-4 and RPN-9 expression patterns suggest these proteins cooperate, particularly in the germline. (**A**) Single and merged UBH-4::EGFP and RPN-9::wrmScarlet signals evidence ubiquitously co-expression of both proteins at different stages (embryo, young adult and adult stages). Scale bar represents 100 µm. Representative images showing endogenous levels of UBH-4 and RPN-9 during spermatogenesis (**B**) and oogenesis (**C**).

If *ubh-4* and *rpn-9* mutations functionally interact, these two genes may be co-expressed in some cells. Taking advantage of *C. elegans* transparency, we aimed to explore both the expression pattern and the endogenous levels of UBH-4 and RPN-9 in a living animal. Thus, we generated a double fluorescent endogenous reporter by Nested CRISPR [29] in the *ubh-4(cer68[ubh-4::EGFP])* background. We introduced the *wrmScarlet* sequence to tag the *rpn-9* locus obtaining the double endogenous fluorescent reporter strain CER620: *ubh-4(cer68[ubh-4::EGFP])*; *rpn-9(cer203[rpn-9::wrmScarlet])II*. Using fluorescence microscopy, we observed that UBH-4 is ubiquitously expressed from embryo to adult stages (Figure 5A). A similar expression pattern was detected for RPN-9 (Figure 5A). Interestingly, we noticed that both proteins were highly expressed in the germline, predominantly in meiotic regions (Figure 5B,C), suggesting a cooperating role for UBH-4 and RPN-9 in germline development.

### 3.7. ubh-4 Deletion and A87D Missense Mutation Enhance the Germline Phenotype of the rpn-9 Deletion Mutant

**Figure 6:**
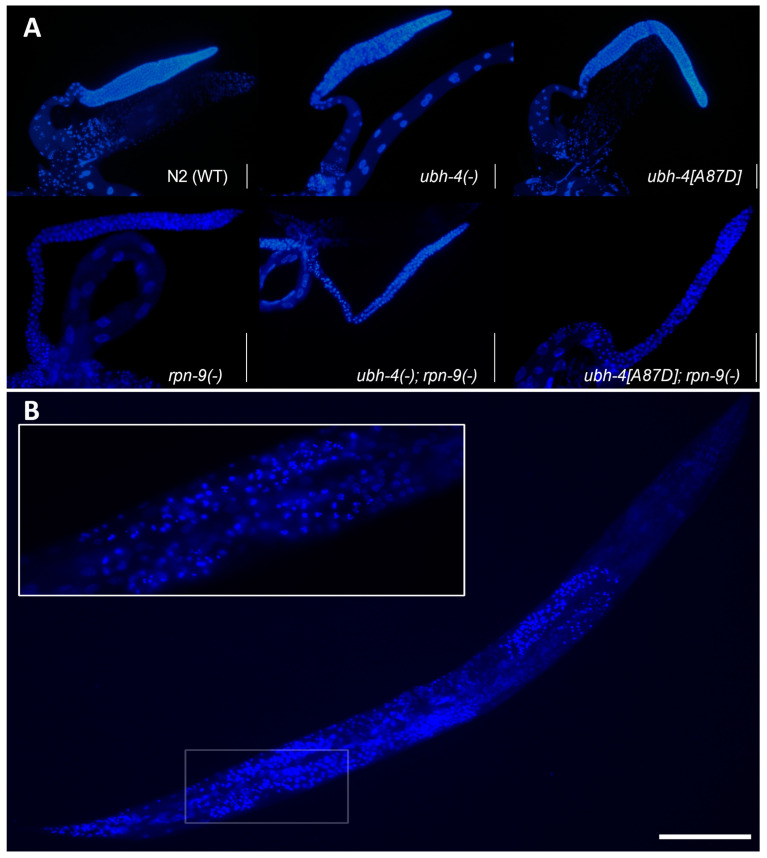
DAPI-stained nuclei in gonads at day one adulthood. (**A**) DAPI staining of dissected gonads from WT, simple and double *ubh-4* and *rpn-9* mutants. (-) indicates deletion. Scale bars represent 50 µm. (**B**) Whole-body DAP staining of a double mutant *ubh-4*(*cer150*); *rpn-9*(*gk104*) showing abundant fragmented nuclei in the germline. The highlighted region is shown at higher magnification (63× lens) at the upper part. Scale bar represents 100 µm.

Since *ubh-4* and *rpn-9* colocalize in the germline and we detected a synthetic reduced brood size between *rpn-9(gk401)* and *ubh-4(cer140)* or *ubh-4(cer198[A87D])*, we studied the dissected germline of these animals after DAPI staining. We observed thinner and smaller germlines in these double mutants than in any single mutant, suggesting a synthetic effect of *rpn-9* and *ubh-4* mutations on germline development (Figure 6A). We detected condensed nuclei in the germline of these double mutants, particularly abundant at the meiotic zone, but we did not quantify these aberrant nuclei since germlines were difficult to compare among themselves (Figure 6B).

### 3.8. Animals Defective in ubh-4 Are Sensitive to the Proteasome Inhibitor Bortezomib

**Figure 7:**
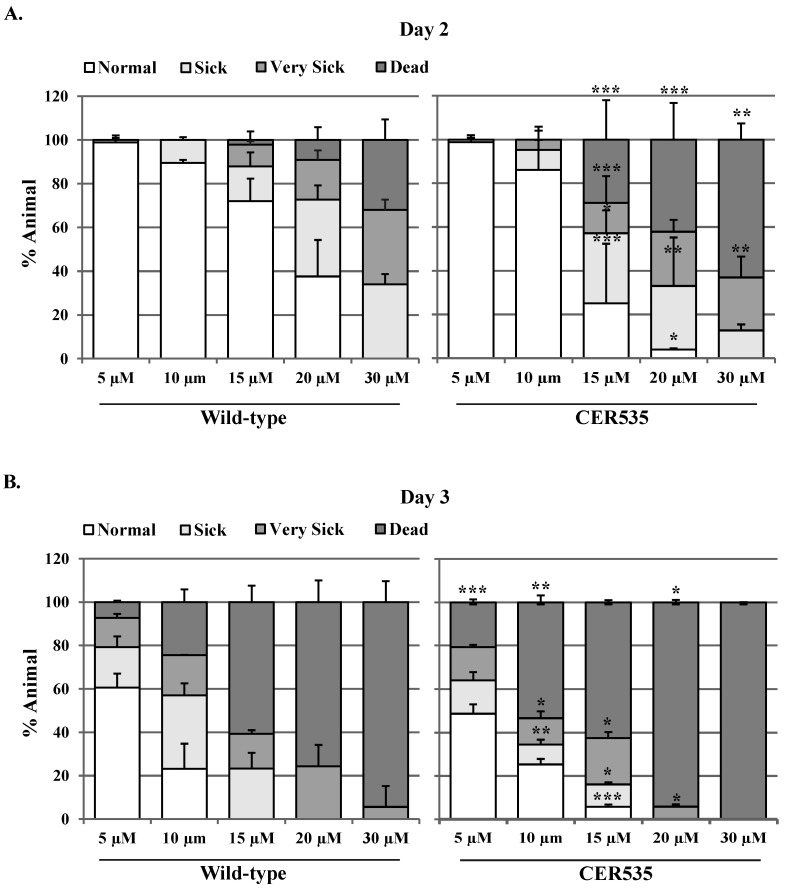
Dose-dependent effect of proteasome inhibitor Bortezomib on *ubh-4* deletion mutant. Graphs represent the health status of both wild-type and CER535 animals under different Bortezomib dosages after two (**A**) and three (**B**) days of exposure at 20 °C. Animals were transferred to Bortezomib-containing plates at the L4 stage and were observed on day 2 and day 3 of adulthood. Animals were scored on basis of mobility and visual appearance as normal, sick (defective movement and appearance), very sick (highly defective movement and appearance), and dead. Results are the mean of three independent experiments (~30 animals in each experiment). Statistical analysis by *t*-test were preformed between wild-type and *ubh-4* mutant (CER535) within each corresponding phenotypic group (normal, sick, very sick, and dead). Error bars, STD, * *p* < 0.05, ** *p* < 0.01 and *** *p* < 0.001.

Since animals without a functional UBH-4 do not display strong phenotypes but sensitivity to the inactivation of *rpn-9*/PSMD13, which is a core proteasome subunit, we asked whether *ubh-4* defective mutants were sensitive to the proteasome inhibitor Bortezomib. Thus, we transferred L4 animals harboring the *ubh-4* deletion allele *cer150* to plates with distinct concentrations of Bortezomib (5 µM, 10 µM, 15 µM, 20 µM, 25 µM, 30 µM) and the effect of this drug was scored at the first days of adulthood. Interestingly, we found that *ubh-4* deletion mutants were sensitive to Bortezomib at days two and three of adulthood (Figure 7).

## 4. Discussion

We established an in vivo model in *C. elegans* to study mutations in the *BAP1* ortholog *ubh-4*. Since *ubh-4* is not essential for viability, *ubh-4* null mutants do not display overt phenotypes and are convenient for RNAi screens in the search of genetic interactions, which, in the context of cancer, may help to investigate potential drug targets. An RNAi screen revealed a synthetic interaction between *ubh-4* and *rpn-9*, a gene encoding a proteasome regulatory subunit. RPN-9 is the ortholog of human PSMD13, a component of the regulatory unit (19S) of the 26S proteasome [37,38]. The moderate inactivation produced by RNAi facilitated the identification of this synthetic interaction since *rpn-9* loss-of-function causes a strong phenotype by itself. The yeast ortholog of *rpn-9* is a non-essential subunit for cell viability [40]. However, in worms, *rpn-9* is crucial for proteasome assembly, protein homeostasis, and proper development [41,42,43]. Moreover, *rpn-9* and other proteasome-forming subunits have been identified as essential for both the lifespan and immunity of germline-deficient animals [44].

Supporting this genetic interaction, endogenous fluorescent reporters for UBH-4 and RPN-9 present an overlapping expression pattern in soma but also in germ cells, where the expression of both genes seems higher. UBH-4 physically interacts with the proteasome and binds the RPN-13 subunit, as well as affects proteasome activity [30]. RPN-9, along with other 19S regulatory subunits, is required a proper proteolytic activity of the proteasome [43]. Since ubiquinated proteins are degraded by the proteasome, the hypothetical mechanisms behind the functional interaction between UBH-4/BAP1 and RPN-9/PSMD13 is unclear. We hypothesize that the inactivation of UBH-4 deubiquitanase activity cooperates with RPN-9 somehow to alter levels of certain proteins that escape the regulated control of degradation in dynamic developmental processes.

The role of the proteasome during germline development, particularly in meiosis, is well-studied [45,46,47,48,49,50]. Thus, the mitotic and meiotic progression of the germline would be a convenient developmental context to further investigate the genetic interaction between *ubh-4* and *rpn-9*. Moreover, as *C. elegans* has orthologs for *BRCA1* and *BRAD1* (*brc-1* and*brd-1*, respectively) [51,52], our model would help to study the mechanisms of controversial functional links between BAP1 and the BRCA1-BRAD1 complex, which play a critical role in DNA repair and homologous recombination, both in mammals [13] and *C. elegans* [53,54]. Since inactivation of the proteasome by RNAi or use of inhibitors produces aberrant nuclei in *C. elegans* germline [43,50] and BAP1 has a regulatory function on ubiquitination of histones [55], genome instability would be the “hallmark of cancer” behind the *BAP1* alterations. Interestingly, although the specific mechanism of carcinogenesis induced by asbestos in MPM is still unclear, it has been proposed that asbestos causes genome instability [56] and chromosomal segregation defects [57]. Given the limited effective therapeutic intervention in MPM [58], and that about 50% of cases of MPM present alteration in *BAP1* [59], the development of animal models such as the one presented here would open new venues to detect vulnerabilities of cells with mutations in *BAP1*.

We mimicked two missense mutations affecting the catalytic region of BAP1, F81V, and A95D. These variants have been associated with diverse types of cancer and both produce structural destabilization of BAP1 [4,60,61]. However, only A95D provoked a phenotype when mimicked in *C. elegans ubh-4*. Previous in vitro studies have suggested that F81V disturbs the catalytic activity of BAP1 [1,4]. However, *ubh-4[F73V]; rpn-9(gk104)* animals did not show abnormal lifespan, body length, or germline development. Therefore, according to our results, F73V missense mutation does not compromise UBH-4 functions. Thus, although *C. elegans* would be a good tool to study the functional impact of mutations and variants of uncertain significance (VUS), there are residues that, despite being conserved, may not present the same functional relevance in worms and humans.

Patients with MPM carry three molecular aberrations in distinct genes on average, more frequently affecting *BAP1*, *NF2*, and *CDKN2A/B* genes [31,60,62]. At lower frequencies, missense mutations in *PSMD13* have also been reported in MPM tumors [60,61] and in whole-exome sequencing studies, including 2680 melanoma, mesothelioma, and clear-cell renal carcinoma samples (Figure S2) [62]. Interestingly, *BAP1* and *PSMD13* genetic alterations do not co-occur in any of the samples reported in these studies (Figure S2), suggesting that the presence of mutations in both genes compromise cell viability, not only in MPM, but also in BAP1-TPDS-related cancer types. Therefore, we hypothesize that the precise targeting of PSMD13 could be a potential therapeutic strategy in *BAP1* mutant tumors.

Here we show that animals carrying *ubh-4* genetic aberrations (complete deletion and A87D missense mutation) are more sensitive to proteasome stresses caused by genetic (*rpn-9* partial or complete silencing) or drug (Bortezomib) targeting. Therefore, gene-based therapeutics focused on disturbing the 19S regulatory complex or *PDMS13* specifically may reduce the proteasome functionality in *BAP1*-affected tumors. With this strategy, it may be possible to re-sensitize MPM tumors to proteasome inhibitors-induced apoptosis (such as Bortezomib), considering that according to a previous study, Bortezomib-sensitive MPM lines display lower proteasome activity [63]. However, since the ubiquitin proteasome system is involved in several key cellular pathways, therapeutic interventions centered on the proteasome would be cautious to avoid adverse secondary effects. Despite Bortezomib failing to demonstrate convincing antitumor activity in clinical trials, either in monotherapy in pretreated patients with MPM or in combination with cisplatin and pemetrexed in the frontline, these clinical trials did not stratify patients based on their genomic profile [64,65,66].

Our study shows that *C. elegans* is a valuable model to explore the functional role of *BAP1* genetic aberrations recurrent in MPM and to investigate drug targets for developing new treatments for BAP1-related MPM.

## Data Availability

Raw data is available upon request.

## Supplementary Materials

The following supporting information can be downloaded at: https://www.mdpi.com/article/10.3390/cells12060929/s1, Figure S1: 20S proteasome levels in *ubh-4* mutants; Figure S2: Genetic alterations in BAP1 and PSMD13 do not cooccur in BAP1-TPDS related cancer. Table S1: 150 genes for the proteasome RNAi sublibrary. File S2: Strains and primers. File S3: CRISPR reagents and injection mixes.
