# Supplementary material for: BAP1 Malignant Pleural Mesothelioma Mutations in Caenorhabditis elegans Reveal Synthetic Lethality between ubh-4/BAP1 and the Proteasome Subunit rpn-9/PSMD13
Source: Cells. 2023 Mar 18;12(6):929. doi: 10.3390/cells12060929 (PMC10047281; doi:10.3390/cells12060929)
Supplement: Supplementary file 1 [file cells-12-00929-s001.zip › Martinez-fernandez_Cells_ Supp files/supplementary figures.pdf]

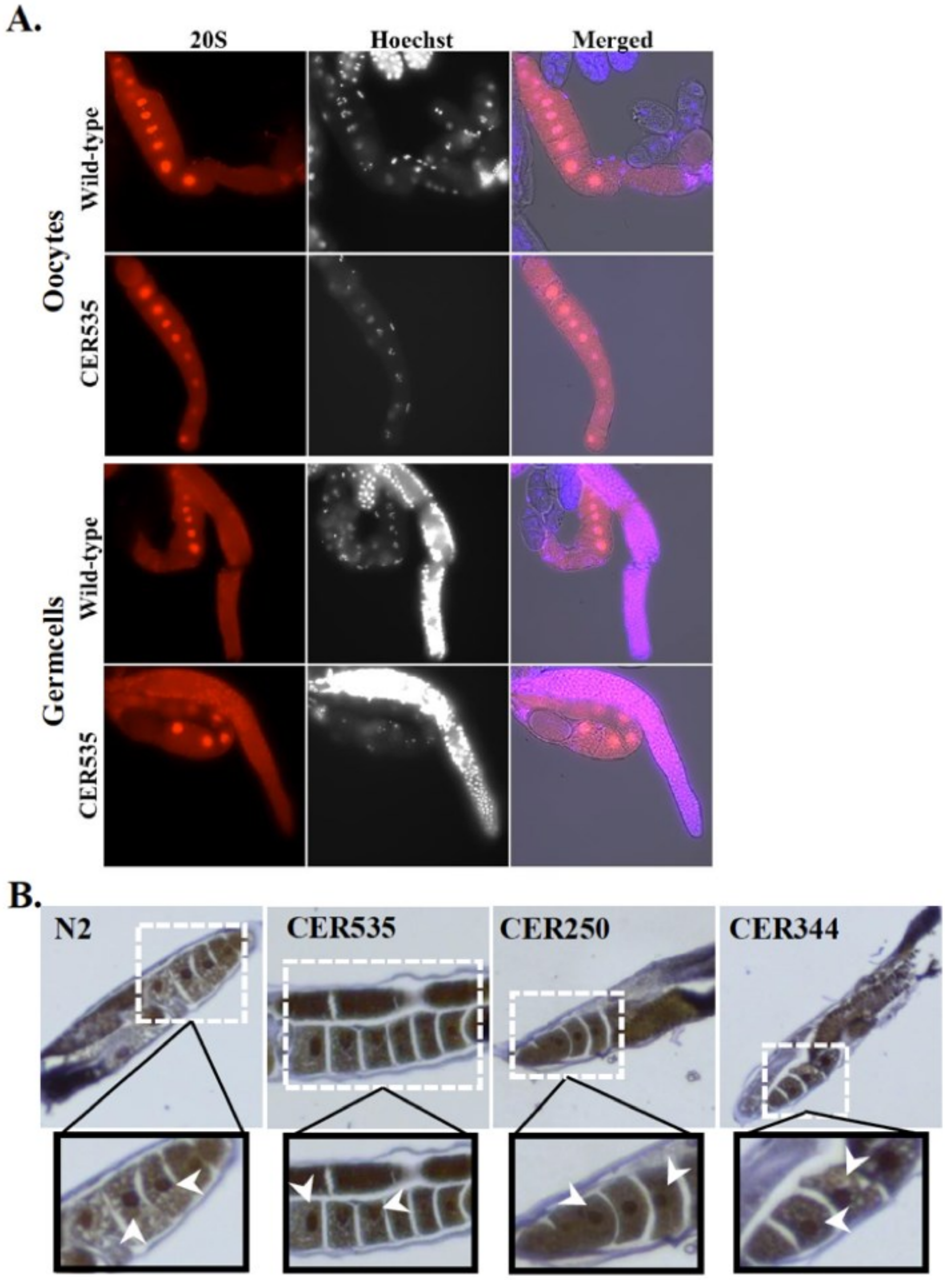

Supplementary Figure S1. *ubh-4* mutations do not affect the tissue expression of the proteosome. A. Representative micrographs of proteasome immunostaining (20S Ab) in dissected oocytes (upper panels) and germlines (lower panels, indicated by white arrowheads) of wild-type and *ubh-4* deletion (CER535 animals). B. Images representing proteasome immunoreactivity of wild-type and *ubh-4* mutant animals in the oocytes upon immunohistochemical staining with 20S Ab (oocytes are indicated by white arrowheads).

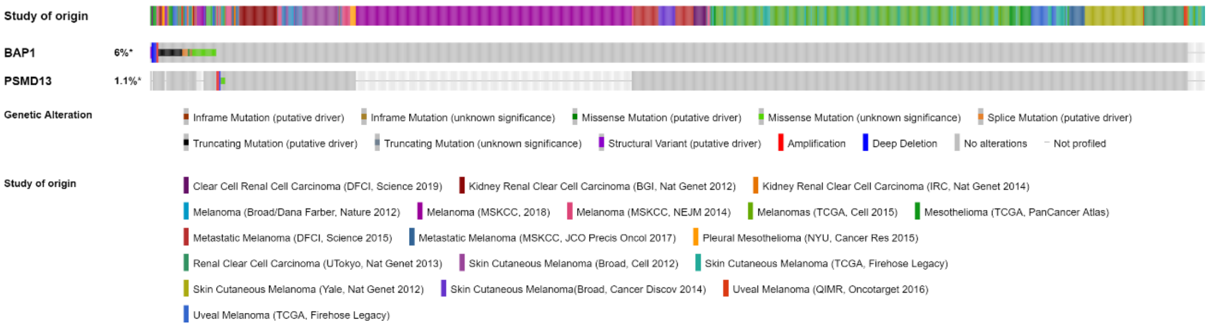

Supplementary Figure S2: Genetic alterations in *BAP1* and *PSMD13* do not cooccur in BAP1-TPDS related cancers. Schematic representation of genetic alteration types affecting *BAP1* or *PSMD13* in a total of 2680 studies including melanoma, mesothelioma, and clear-cell renal carcinoma samples. Data collected from cBioportal [57,62].
